# Supplementary material for: Metabolic Profiling and Transcriptional Analysis of Carotenoid Accumulation in a Red-Fleshed Mutant of Pummelo (Citrus grandis)
Source: Molecules. 2022 Jul 19;27(14):4595. doi: 10.3390/molecules27144595 (PMC9324369; doi:10.3390/molecules27144595)

Figure S1. GC/MS total ion chromatograms of two different tissues of R-HJH and wild-type HJH fruits. A, flavedo of HJH; B, albedo of HJH; C, flavedo of R-HJH; and D, albedo of R-HJH.

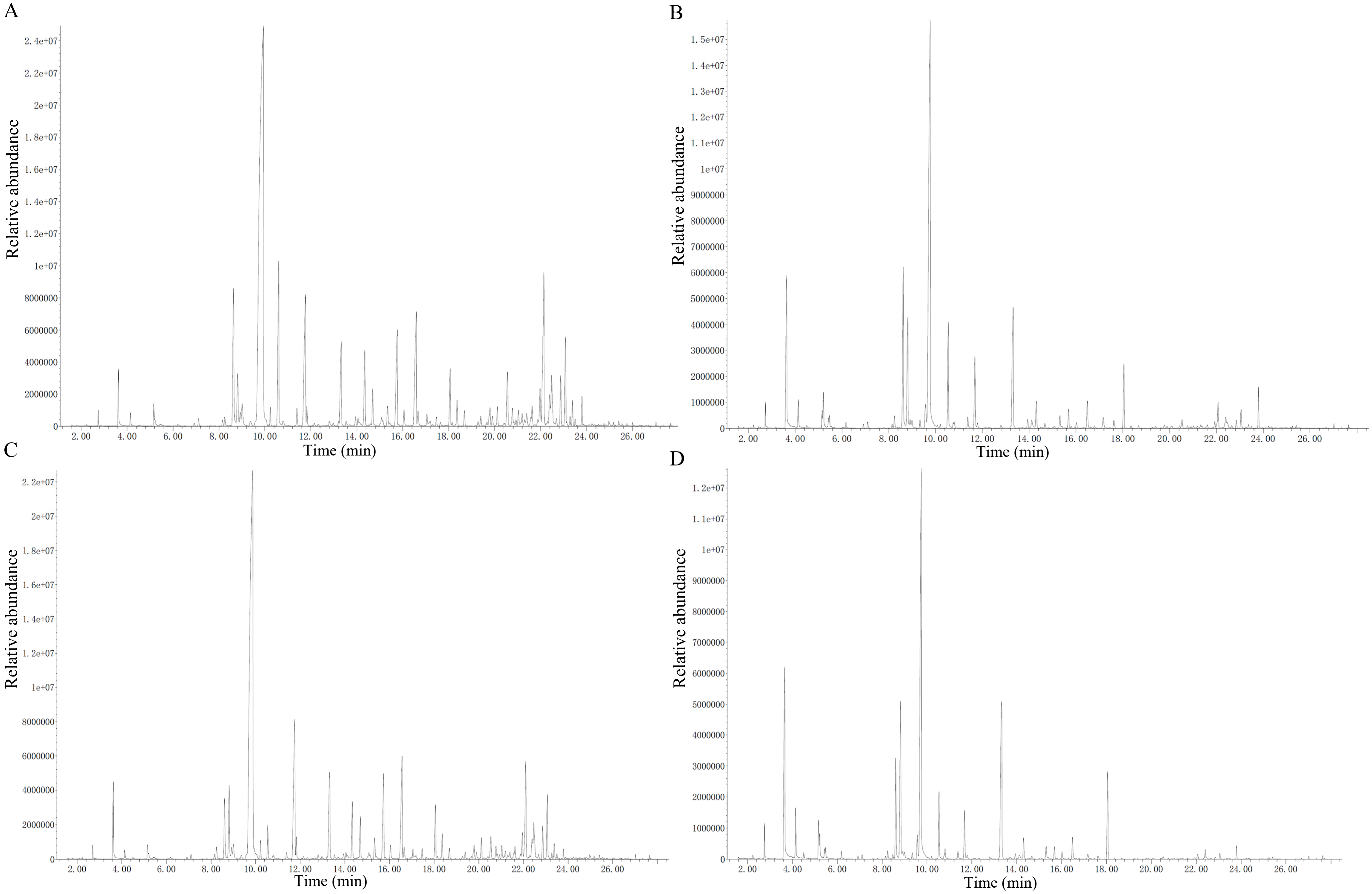

Figure S2. Volcano map of differentially expressed genes. Each point in the figure represents a gene, and the red and green dots indicate that the expression level of DEGs is upregulated and downregulated, respectively.

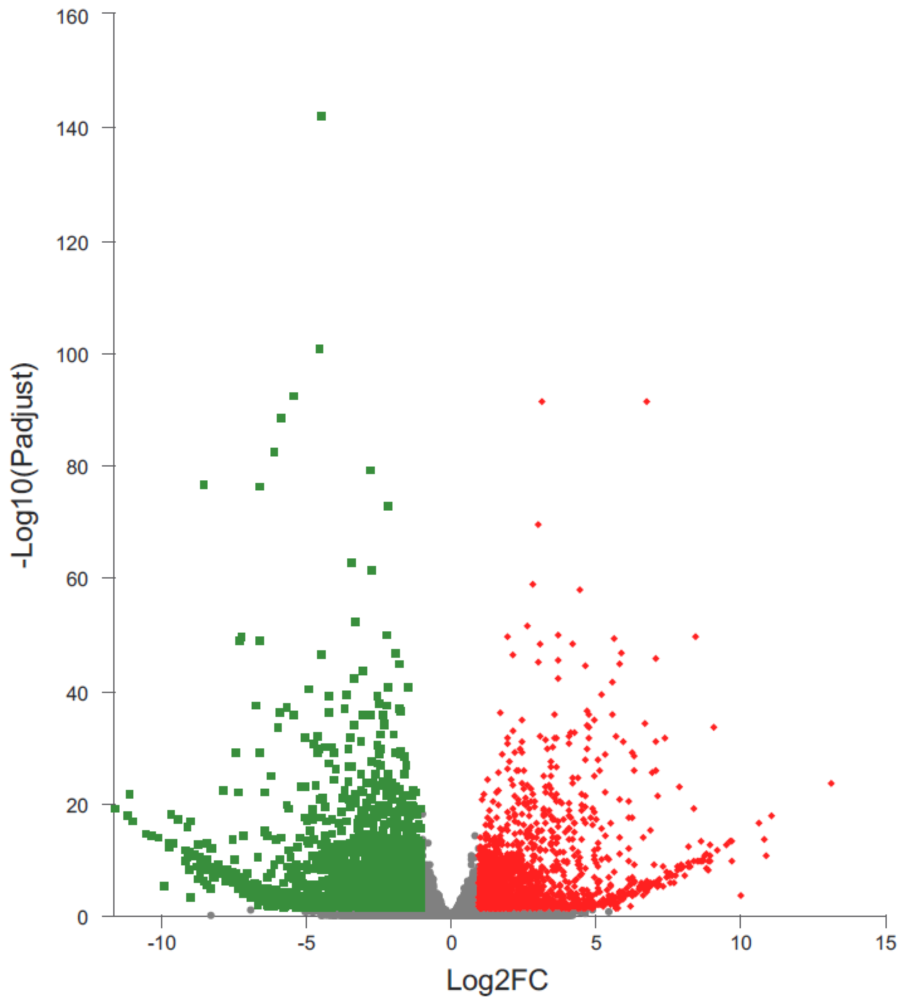

Supplement: Supplementary file 1 [file molecules-27-04595-s001.zip › Figures S1-S2.pdf]
