# Supplementary material for: Metabolic Profiling and Transcriptional Analysis of Carotenoid Accumulation in a Red-Fleshed Mutant of Pummelo (Citrus grandis)
Source: Molecules. 2022 Jul 19;27(14):4595. doi: 10.3390/molecules27144595 (PMC9324369; doi:10.3390/molecules27144595)
Supplement: Supplementary file 1 [file molecules-27-04595-s001.zip › Table S1.pdf]

Table S1 quality analysis of transcriptome sequencing data

sample name	Raw reads	Clean reads	Error rate %	Q20 %	Q30 %	GC content %	Contrast ratio%
**HJH Albedo 1**	41725292	41301096	0.0257	97.8	93.38	45.24	88.87
**HJH Albedo 2**	50405050	49618582	0.0256	97.85	93.52	45.1	90.96
**HJH Albedo 3**	44951404	44363436	0.0262	97.59	92.9	44.9	92.05
**R-HJH Albedo 1**	52446264	51948746	0.0257	97.79	93.38	44.52	93.12
**R-HJH Albedo 2**	49635476	49171630	0.026	97.7	93.14	44.83	92.7
**R-HJH Albedo 3**	47521580	47099778	0.0257	97.79	93.36	44.93	90.69
