# Supplementary material for: Metabolic Profiling and Transcriptional Analysis of Carotenoid Accumulation in a Red-Fleshed Mutant of Pummelo (Citrus grandis)
Source: Molecules. 2022 Jul 19;27(14):4595. doi: 10.3390/molecules27144595 (PMC9324369; doi:10.3390/molecules27144595)
Supplement: Supplementary file 1 [file molecules-27-04595-s001.zip › Table S5.pdf]

Table S5 DEGs involved in carotenoid accumulation

Gene_id	Gene name	FC(LA/CK)	Log2FC(LA/CK)	Pvalue	Padjust	Significant	Regulate
**Cg2g044950**	NCED1, 9-cis-epoxycarotenoid dioxygenase	11.132	3.476579	4.76E-15	1.55E-13	yes	up
**Cg3g011400**	ZDS1, Zeta-carotene desaturase	0.417	-1.26349	0.00016	0.000798	yes	down
**Cg3g021360**	ABA 8'-hydroxylases 4	3.12	1.641578	0.000301	0.001391	yes	up
**Cg5g002300**	CHYb, β-carotene hydroxylase 2	0.489	-1.03261	0.011767	0.032431	yes	down
**Cg5g034750**	β-carotene isomerase D27	6.468	2.69343	0.004052	0.013295	yes	up
**Cg5g044040**	CCD7, Carotenoid cleavage dioxygenase 7	0.457	-1.13093	5.5E-05	0.000311	yes	down
**Cg6g001440**	Protein LUTEIN DEFICIENT 5	2.202	1.138645	6.61E-07	5.68E-06	yes	up
**Cg6g016720**	PSY, phytoene synthase	2.233	1.159279	0.000204	0.000988	yes	up
**Cg6g020290**	ABA 8'-hydroxylases 1	0.137	-2.871443	1.06E-09	1.53E-08	yes	down
**Cg8g004210**	ABA β-glucosyltransferase	2.179	1.123607	0.001537	0.005773	yes	up
**Cg8g022720**	ABA 8'-hydroxylases 2	0.319	-1.649825	0.000835	0.003414	yes	down
**CgUng001590**	LCYE, Lycopene epsilon cyclase	4.167	2.058897	4.41E-10	6.78E-09	yes	up
